# Candidate Genes Associated with Survival Following Highly Pathogenic Avian Influenza Infection in Chickens

**DOI:** 10.3390/ijms251810056

**Published:** 2024-09-19

**Authors:** Wioleta Drobik-Czwarno, Anna Wolc, Callie R. Petal, Katarzyna Miedzinska, Jack Dekkers, Janet E. Fulton, Jacqueline Smith

**Affiliations:** 1 Department of Animal Genetics and Conservation, Institute of Animal Science, Warsaw University of Life Sciences, 02-787 Warsaw, Poland; wioleta.drobik@gmail.com; 2 Department of Animal Science, Iowa State University, Ames, IA 50011, USA; awolc@iastate.edu (A.W.); jdekkers@iastate.edu (J.D.); 3 Hy-Line International, P.O. Box 310, 2583 240th St., Dallas Center, IA 50063, USA; jfulton@hyline.com; 4 The Roslin Institute and Royal (Dick) School of Veterinary Studies R(D)SVS, University of Edinburgh, Easter Bush, Midlothian EH25 9RG, UK; lonelycarp@hotmail.com (C.R.P.); kasia.miedzinska@roslin.ed.ac.uk (K.M.)

**Keywords:** highly pathogenic avian influenza, whole-genome sequencing, genetic variation, GWAS, resistance, chicken

## Abstract

Highly pathogenic strains of avian influenza (HPAI) devastate poultry flocks and result in significant economic losses for farmers due to high mortality, reduced egg production, and mandated euthanization of infected flocks. Within recent years, HPAI outbreaks have affected egg production flocks across the world. The H5N2 outbreak in the US in 2015 resulted in over 99% mortality. Here, we analyze sequence data from chickens that survived (42 cases) along with uninfected controls (28 samples) to find genomic regions that differ between these two groups and that, therefore, may encompass prime candidates that are resistant to HPAI. Blood samples were obtained from survivors of the 2015 HPAI outbreak plus age and genetics-matched non-affected controls. A whole-genome sequence was obtained, and genetic variants were characterized and used in a genome-wide association study to identify regions showing significant association with survival. Regions associated with HPAI resistance were observed on chromosomes 1, 2, 5, 8, 10, 11, 15, 20, and 28, with a number of candidate genes identified. We did not detect a specific locus which could fully explain the difference between survivors and controls. Influenza virus replication depends on multiple components of the host cellular machinery, with many genes involved in the host response.

## 1. Introduction

Highly pathogenic avian influenza (HPAI) can evolve from H5 and H7 strains of lowly pathogenic avian influenza (LPAI) [1] and has multiple detrimental effects on the global poultry industry, causing high levels of mortality, obligatory euthanasia, and closing export opportunities [2]. During the widespread H5N2 outbreak in the US in 2015, more than 43 million birds in 15 states had been destroyed within 3 months of the start of the outbreak, including nearly 30 million in Iowa alone, the largest egg producing state in the US. In the Midwest, the average egg price increased 120% [3]. Costs to the US economy (direct and indirect) were estimated to be around $3.3 billion USD [4]. The virus was determined to be a reassortment of clade 2.3.4.4 of the goose/Guangdong/1996 (GS/GD/96) H5 lineage of HPAIV (contributing gene segments: PB2, PA, HA, M, and NS) and North American LPAI strains (contributing gene segments: PB1, NP, and NA) [5]. This outbreak resulted in a mortality rate of >99%. However, a small proportion of birds (<0.1%) on commercial sites in Iowa were found to have survived up to 4 weeks post-infection with continued exposure to virus before they were euthanized. With host genetics known to be involved in determining pathological outcome [6], HPAI survivors may contain natural genetic mutations that make them either resistant to the virus or able to withstand morbidity/mortality associated with infection. Therefore, DNA samples from these survivors and non-infected age- and genetics-matched controls were obtained with the aim of identifying genetic regions associated with resistance to HPAI. Identifying these mutations would be extremely valuable, not only because it would provide a mechanism for using natural genetic variation to breed chickens that are more resistant to HPAI, but also because this will provide information on mechanisms of avian influenza (AI) infection and resistance for development of vaccines or other viral control measures. In our previous study using a 600 K genotyping panel, several potentially interesting regions were identified, but there was no single strong signal that could define genetic differences between survivors and controls. Based on those results, resistance to AI was presumed to be a complex trait determined by many genes [7]. Using a SNP (single nucleotide polymorphism) panel is a good strategy for detecting quantitative trait loci (QTL), but it also has its limitations: of 1.2 billion base pairs in the chicken genome, only 600,000 are surveyed with this SNP panel [8]. Any novel mutation(s) responsible for resistance are likely not included on the chip. This limitation can be overcome by whole-genome sequencing, which provides information on every position in the genome. The whole-genome sequence also provides information on other genetic variants that are not well captured by the SNP chips, including copy number variants (CNVs), deletions, reversions, and recombinations.

The objective of this study was to use these unique samples to compare whole-genome-sequencing data from survivor and control birds to identify regions, and potentially genes, in the chicken genomes that are associated with resistance to HPAI. The need to understand the host genetics underlying resistance has never been more pressing, as HPAI continues to spread globally [9,10], not just in poultry but also infecting many different bird species [11,12,13], as well as crossing over to several mammalian species [14,15,16]. The chance of zoonotic spread also remains a huge risk [17,18], as viruses continue to mutate and increase their host range.

## 2. Results

### 2.1. Identification of Single Nucleotide Polymorphisms (SNPs)

**Figure 1:**
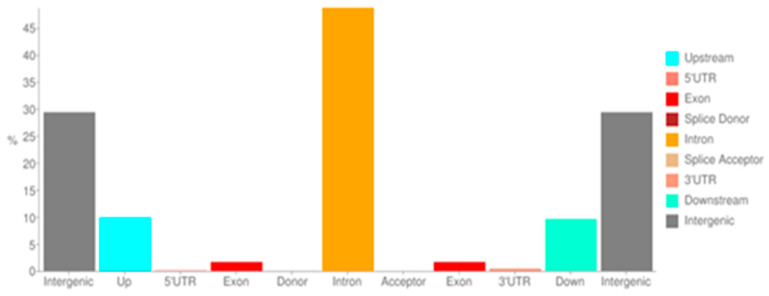
SNP variant distribution according to the Ensembl (v86) transcript database.

In total, 10,468,843 SNPs were retained after filtering. The number of effects by type and region are shown in Figure 1.

The majority of variants were located in introns (over 45%) and intergenic regions (around 30%), with a small proportion of variants in exons and 5′ and 3′ UTR regions. The potential impacts of the variants were based on simple estimation of putative functional effects. The number of effects found is summarized in Table 1A. The majority of detected SNPs (over 98.5%) were within the ‘modifier’ category, which refers to changes outside the coding regions, so their impact on protein function is therefore more difficult to estimate. However, as all variants within regulatory regions, such as transcription factor binding sites or non-coding RNAs, also fall into this category, their importance should still be considered. High- and moderate-impact categories result in frame shifts, addition/deletion of stop codons and codon change, deletions, or insertions. These changes, which accounted for less than 0.5% of all annotations, were analyzed in more detail. The number of variants by functional class is shown in Table 1B. The non-sense classification was assigned to point mutations that result in the creation of a new stop codon, missense was assigned to point mutations that result in an amino acid change but not a new stop codon, while the silent category was assigned to point mutations that result in a codon change but not an amino acid change or a new stop codon.

### 2.2. Association Analysis

**Figure 2:**
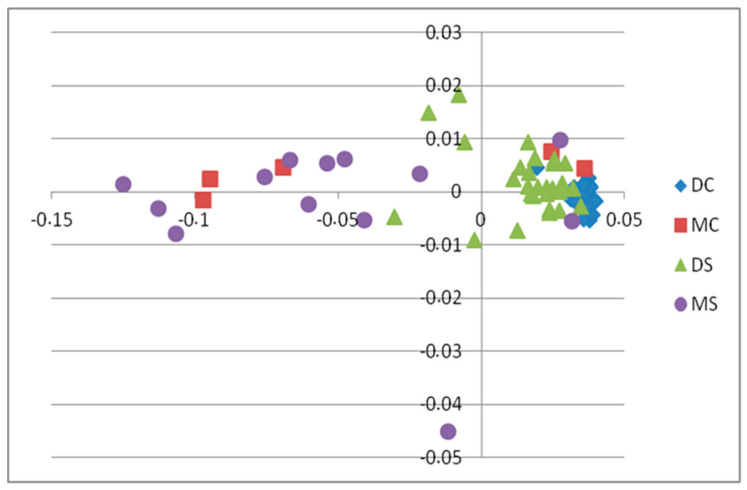
First two principal components from multi-dimensional scaling analysis. The first letter is an indication of sample origin (company), while the second letter points to cases (S = survivors) or controls (C).

In total, 58,756 SNPs were retained after pruning and used in an MDS (multi-dimensional scaling) analysis. The samples were obtained from two different commercial farms (designated ‘D’ and ‘M’). The distribution of cases and controls was dependent on the sequencing quality of the DNA obtained from survivors and is shown in Figure 2.

**Figure 3:**
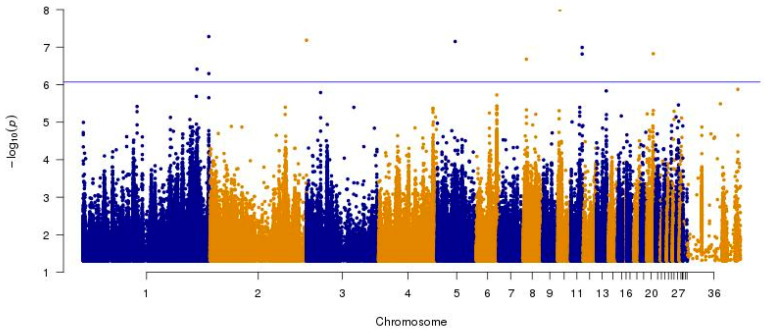
Manhattan plot for case–control association tests (*n* = 70). Each dot corresponds to a SNP, and its colour indicates its chromosomal location.

Lowest sequencing quality was observed for survivor samples from source M (MS), which is the likely cause of their differentiation from the other samples. From the 10,468,843 total SNPs, 6,263,749 were found to be informative after quality control (MAF > 0.05, Marker Genotyping Rate > 0.8). A basic case–control test in Plink was performed. The genome-wide significance threshold was set as 0.05 divided by the number of independently segregating SNPs, which was 8.5 × 10^−7^. In total, 10 SNPs exceeded the genome-wide significance threshold (Figure 3).

The location of significant SNPs and details of the genes they overlap are shown in Table 2. Significant SNPs included intronic variants in *SMPD1* on Chr 1, *SYNJ2BP* on Chr 5, *ADAMTSL2L* on Chr 8, *DAPK2* on Chr 10, and *ANGPT4* on Chr 20, along with a variant upstream of the *APBB1* gene on Chr 1 and one upstream of *NRBP2* on Chr 2. Due to variable coverage, sequence quality, and clustering of samples, further investigation was performed with correction based on the first two principal components from the MDS analysis shown in Figure 2. A logistic model was used with the missing genotype rate per marker set to 0.2 and the minimum minor allele frequency (MAF) set to 0.05. However, the analysis performed on these 6,168,273 variants resulted in no significant associations.

### 2.3. SNPs Selected Based on Potential Functional Impact

A number of regions had previously been identified in a study based on the 600 K Affymetrix SNP chip using a larger sample size (585 genotyped samples) [7,19]. All SNPs detected in the current study were selected for further analysis based on their predicted high or moderate functional impact, a GWAS *p*-value < 0.0001, and falling within 2 Mb of regions identified in our 600 K genotyping study. All the selected variants are shown in Table 3 and included mutations in the *CAV2* gene on Chr 1, *NR3C2* on Chr 4, *CHRD* on Chr 9, and *RAN* on Chr 15.

### 2.4. Correlation between Whole Genome Sequences and 600 K Genotypes

**Figure 4:**
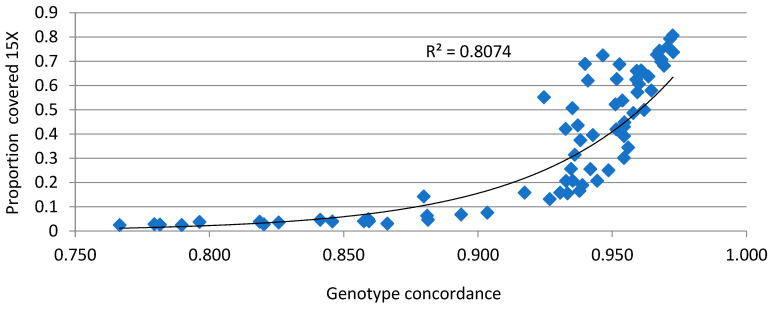
Genotype concordance between genotypes called based on whole-genome sequence and the 600 K SNP panel and % of the genome covered at minimum 15X.

For the 70 samples that were sequenced, 600 K Affymetrix^®^ Axiom^®^ SNP panel [8] genotype data were also available. In total, 420,458 high quality SNPs were available from the 600 K panel genotyping. Correlation of allele dosage (0,1,2) was used to assess the concordance between whole-genome sequencing (WGS) data and 600 K data (Supplementary Table S1). Liftover (Affy liftover + UCSC liftover) was used to convert the 600 K data (based on the Galgal4 chicken reference genome) to Galgal5 co-ordinates. The following thresholds were used for quality control of each dataset: MAF > 0.01 and Marker Genotyping Rate > 0.8. In total, 307,482 SNPs were retained after filtering and were found to share genome coordinates and the reference allele with the whole-genome sequence data. Genotype concordance (GC) was calculated as the proportion of array-derived genotypes that were the same as the sequence-derived genotypes over all non-missing genotypes of the sequence-derived genotypes. This was found to be relatively high for the majority of samples and highly correlated with % of coverage of 15X with an exponential trend (Figure 4). Subsequent investigation determined that the observed low correlations were the result of calling heterozygotes as reference homozygotes due to lack of the alternate allele for regions with low coverage. This was observed mainly for samples for which the proportion of 15X coverage was lower than 10% and for which the mean coverage was lower than 20X.

### 2.5. Imputation of Missing Genotypes

**Figure 5:**
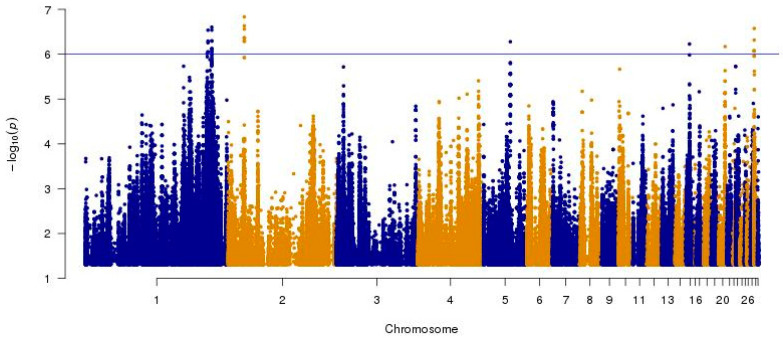
Genome-wide association results after imputation of missing genotypes. Each dot corresponds to a SNP, and its colour indicates its chromosomal location.

Imputation of missing genotypes was performed on the dataset. The imputation accuracy was calculated for 1% of the SNPs randomly selected across the genome. The genotype concordance for imputed missing genotypes was based on 12 samples that were selected to represent the full range of sequence quality (Supplementary Table S2). The total number of sites validated was 58,833. Association analysis for cases and controls was performed after imputation of missing genotypes and yielded 26 significant SNPs, but none of these SNPs were found to overlap with the pre-imputation analysis. The strongest signals were obtained on chromosomes 1, 2, 5, 15, 20, and 28 (Figure 5). Genes that overlap these significant regions include an lncRNA (*ENSGALG00000068037*) on Chr1, *AKAP9* on Chr2, *SLC25A21* on Chr5, lncRNAs on Chr15 (*ENSGALG00000064574*) and Chr20 (*ENSGALG00000049648*), and a novel gene (*ENSGALG00000047550*) on Chr28 (Table 4).

### 2.6. Identification of Insertions and Deletions (INDELs) from Whole Genome Sequence Data

**Figure 6:**
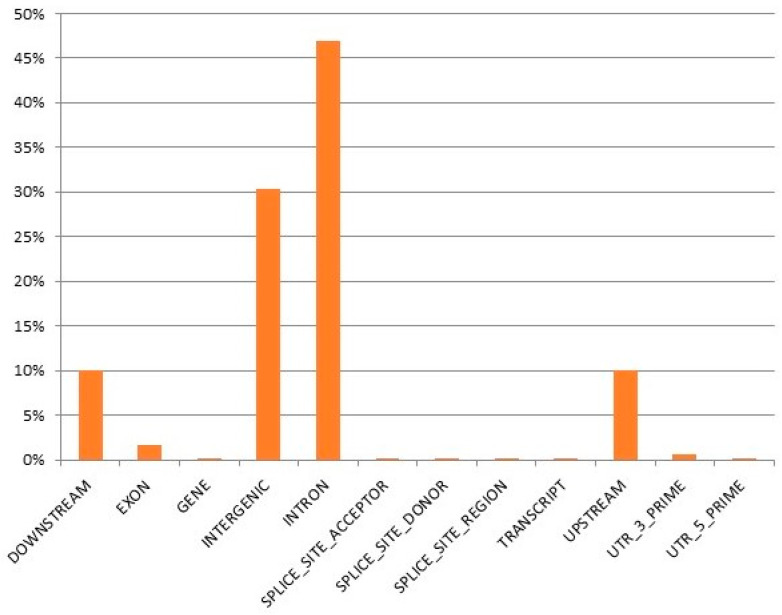
Annotation of INDELs by genetic region.

After filtering, 4,143,372 INDELs were retained, and their impacts and regional distributions are illustrated in Figure 6 and Table 5. The majority of insertions and deletions were observed in introns. In total, around 50% of these were located in either intergenic regions or were located downstream or upstream of a gene. Insertions and deletions were also tested for frequency differences between cases and controls (Table 6). The most significant of these included INDELs in intronic regions of *CORO2B* (Chr10), *RNFT1* (Chr19), and *PALM2* (ChrZ), along with INDELs upstream of *ANP32A* (Chr10) and *PRCC* (Chr25). All variant impacts and gene functions were determined based on the Ensembl v86 database and gene ontology terms.

### 2.7. Functional Enrichment of Candidate Genes

No particular pathways were identified as significant when the candidate genes identified in this study were examined using QIAGEN Ingenuity Pathway Analysis. Available online: https://digitalinsights.qiagen.com/products-overview/discovery-insights-portfolio/analysis-and-visualization/qiagen-ipa/ (accessed on 26 June 2024).This is most likely due to the small number of genes examined, although it may well be that none of the identified genes belong to the same biological networks. However, when the genes were analyzed for functional over-representation, it was interesting to see that ‘viral life cycle’ and ‘sphingolipid metabolism’ [20] showed a significant *p*-value when using WebGestalt, although the FDR was not significant (Supplementary Figure S1). As well as being involved in the immune system, genes involved in these pathways could potentially be of importance for both growth and egg production—factors which may be affected after infection by avian influenza. Sphingolipid metabolism has been shown to be associated with muscle development and energy metabolism [21,22], as well as follicular development [23] and oocyte quality, in humans [24]. Likewise, genes associated with viral life cycle may also be associated with growth-signaling pathways [25] and hormonal disruption [26]. These genes thus warrant further investigation.

### 2.8. Genotyping of Elite Commercial Lines

Frequencies of alleles from 130 selected SNPs (Supplementary Table S3) were obtained from the 2017 and 2018 generations of four elite White Leghorn lines used to generate commercial birds which produce eggs for consumption (Table 7), as these lines were the most recent generations of the elite lines that were considered to be the most relevant as the pure line source of birds in the field outbreak at the time of testing. Not all SNPs were segregating in all lines, and SNP allele frequencies varied between lines. The primary purpose of this genotyping was to determine within which lines these SNP were segregating and could thus be possible targets to improve HPAI resistance in subsequent generations. All 130 SNPs showed segregation in at least one line, and 55% of the SNP-by-line combinations showed within-line segregation. This shows that each of these SNPs has the potential for selection within at least one elite line if shown to be associated with HPAI resistance.

## 3. Discussion

Having access to a unique set of samples from commercial chickens that were able to survive an outbreak of HPAI H5N2 in the face of >99% mortality allowed us to examine the chicken genome for regions potentially conferring resistance to infection or, at least, the effects of such an infection. GWAS of survivor birds compared to age- and genetics-matched controls identified six chromosomes (Chrs 1, 2, 5, 8, 10, 11, 20) with regions that contained significant SNPs.

Genes underlying these regions include genes which have biological functions that are potentially relevant for avian flu survival/susceptibility. The *DAPK2* (death-associated protein kinase 2) gene on Chr10 is a calcium/calmodulin-dependent serine/threonine kinase involved in multiple cellular signaling pathways that trigger cell survival, apoptosis, and autophagy [27,28]. It has previously been shown to be a human–host factor that is required for influenza virus replication [29]. The *NRBP2* (nuclear receptor-binding protein 2) gene on Chr2 enables protein serine/threonine kinase activity and is involved in the negative regulation of macroautophagy [30]. It is also interesting to note that its paralogous gene, *NRBP1*, is involved in the early steps of viral replication [29]. Significant variation was also found within the *SMPD1* (sphingomyelin phosphodiesterase 1) gene on Chr1. The secreted form of the protein encoded by this gene is known to be increased in response to stress and inflammatory mediators such as IL1B, IFNG, or TNF, as well as upon infection with bacteria and viruses such as SARS-CoV-2 [31]. Similarly, the *APBB1* (amyloid beta precursor protein binding family B member 1) gene on Chr1 has previously been identified as a hub gene during response to SARS-CoV-2 infection in humans [32]. Significant variation within the *ANGPT4* (angiopoietin 4) gene on Chr20 was also indicated to be associated with resistance to AI, with increased levels of ANGPT4 previously shown to be related to acute respiratory distress syndrome and severity of SARS-CoV-2 infection in humans [33].

In order to improve signals identified by GWAS, imputation was carried out using whole-genome sequence data from twelve samples (as detailed in Supplementary Table S2). Significant regions (Chrs 1, 2, 5, 15, 20, 28) showed some discordance with the regions and genes that were identified to be significantly associated with HPAI resistance pre-imputation. Assuming that rare or novel alleles may be contributing to the resistant phenotype, it could be that, in this case, imputation is not helpful in discerning the associated mutations, as these rare alleles may not be represented in the samples used for imputation. Some protein-coding genes were found to locate in the identified regions following imputation. These included the *AKAP9* (A-kinase anchoring protein 9) gene on Chr2 and the *SLC25A21* (solute carrier family 25 member 21) gene on Chr5. Neither gene has biological function that is obviously related to viral pathogenesis, but, again, related paralogues encode proteins which have previously been associated with influenza virus: AKAP10 binds to polymerase PA-PB1 [34], while AKAP13 is involved in early steps of viral replication [17], and SLC25A25 facilitates host–cell resistance to lethal influenza challenge [35].

An analysis of variation in INDELs was also carried out, which highlighted four regions of the genome that could be contributing to the survival phenotype. Genes in these regions include *CORO2B*, *ANP32A*, *RNFT1*, *PRCC*, and *PALM2*. The *PRCC* (proline-rich mitotic checkpoint control factor) gene regulates cell cycle progression [36] and may play a role in pre-mRNA splicing [37]. INDELs associated with HPAI survival are located upstream of this gene. The 57 bp deletion on chromosome 19 is located within the *RNFT1* gene (ring finger protein, transmembrane 1), which is involved in protein auto-ubiquitination and positive regulation of the ERAD pathway [38]. Functionality of the *PALM2* (paralemmin 2) gene on Chr Z has yet to be determined. A significant indel on chromosome 10 (19,246,320) is located within the *CORO2B* (coronin 2B) gene, which influences cytoskeletal plasticity [39]. More interestingly, this indel is 20 kb upstream of the adjacent *ANP32A* (acidic nuclear phosphoprotein 32 family member A) gene (chromosome 10, 19,263,595–19,281,765 bp). ANP32A is required for replication by avian influenza viruses and is a candidate host target for novel antivirals [40,41].

We also examined genes which were predicted to contain deleterious functional mutations. These included the *CAV2*, *CHRD*, *NR3C2*, and *RAN* genes. Two of these genes—*CAV2* and *RAN*—have potentially relevant biological functions. The *RAN* (GTP-binding nuclear protein) gene is located on Chr15, and its human protein orthologue is involved in the Influenza Viral RNA Transcription and Replication super-pathway (https://pathcards.genecards.org/; accessed on 20 January 2023). Various RAN-binding proteins have also been implicated in influenza infection in humans: RANBP3 (RAN-binding protein 3) is involved in viral RNP nuclear export [42]; RANBP6 (RAN-binding protein 6) binds to the PA polymerase [22], and siRNA knockdown of RANBP9 (RAN-binding protein 9) decreases viral replication [43]. A missense mutation in the *CAV2* (Caveolin-2) gene was also detected and found to be present in the dbSNP database with ID rs734852275. However, the associated Glu→Ala amino acid change is predicted to be tolerated. A closely related gene, *CAV1*, is known to influence human influenza A virus propagation [44], and CAV1 has been found to negatively regulate influenza virus replication in mouse fibroblasts, with depletion of CAV1 causing an increase in infectious production of more than two orders of magnitude [45], which suggests a similar mechanism in poultry, at least in vitro.

The commercial birds of the field challenge were produced from planned crosses of four elite lines, as routinely performed to utilize hybrid vigor. Thus, while genome variation (including for SNPs) can segregate in commercial birds, it is possible that some or all of the originating pure lines are fixed, albeit for the different alleles. If selection were to be initiated for specific alleles, there would need to be segregation for those alleles within the pure line. The observation of segregation of each of the analyzed SNPs in different lines and breeds shows that these are potentially usable targets for selection and can inform a selective breeding strategy for experimental purpose and biological validation of the effects. To evaluate the risk of adverse effects from selection for HPAI resistance on other economically important traits, these SNPs should also be examined to determine if they have negative associations with other traits in the elite parental lines such as egg production and egg quality (using progeny means for males).

The next logical step in this research is to collect and analyze samples from additional HPAI outbreaks to see if the genomic regions/genetic variants identified in this study are confirmed in other populations and indeed in response to challenge by other strains of the AI virus. It does seem likely that birds mount different host responses to infection with different strains, as has been indicated by previous genetic analysis [7] and transcriptomic studies [46,47,48]. In fact, some of the genes indicated in this study have been shown to have altered gene expression in some of these earlier papers.

## 4. Materials and Methods

### 4.1. Sample Collection

All samples were taken from mature female Hy-Line W36 commercial production chickens, from both infected and uninfected premises in Iowa, USA. All blood samples (50 µL) were collected onto FTA elute cards (part number: WB120410, GE Healthcare, Little Chalfont, Buckinghamshire, UK). Due to legislated biohazard control measures, the FTA cards from survivors were autoclaved. Survivor samples were obtained from two commercial production companies (designated ‘D’ and ‘M’) which had a few (<50) birds still alive four weeks after initial avian influenza (AI) diagnosis, with all other birds having died (>250,000 per barn). The control samples were obtained from birds in non-infected facilities from the same two production companies that were hatched at the same time and from the same hatchery source, thus ensuring common genetics.

### 4.2. Sample Preparation

From each sample, a 1.2 mm punch of FTA card with blood sample was obtained and placed into 100 µL of extraction buffer, heated to 56 °C for 15 min, then heated to 99 °C for 10 min, and centrifuged to collect eluted DNA. A 1 µL volume of eluate for each sample was analyzed by PCR. Quality control of the final DNA showed small amounts of fairly low-quality DNA (as determined using a Tapestation 4200—Agilent, Stockport, UK), especially from the autoclaved survivor samples. To improve the quality and quantity of the DNA samples, we carried out whole-genome re-amplification using the Reply-g kit (Qiagen, Hilden, Germany, 150025), followed by purification using the Genomic DNA Clean & Concentrator kit (Zymo, Irvine, CA, USA, D4064). The samples with the lowest concentration were run in duplicate, then pooled and purified. The DNA concentrations were determined using a Qubit 4 Fluorometer. The samples were prepared using the dsDNA HS kit (Q32850; Thermofisher, Paisley, UK), using the manufacturer’s protocol. For accuracy, all samples were measured in duplicate, and the average of the two measurements was used for further application. A Tapestation 4200 was used to determine the quality and size of the DNAs. For DNA visualization, genomic DNA screen tapes were applied, along with reagents (Agilent 5067–5365 and 5067–5366). Final DIN numbers were between 6 and 8 for all samples.

### 4.3. Whole Genome Sequencing

Seventy samples (42 cases; 28 controls), which were previously genotyped with the 600 K Affymetrix chicken SNP chip [8], were sequenced on a HiSeqX platform at Edinburgh Genomics (Edinburgh, UK). Next Generation sequencing libraries were prepared using Illumina SeqLab specific TruSeq Nano High Throughput library preparation kits (Illumina, Cambridge, UK), in conjunction with the Hamilton MicroLab STAR and Clarity LIMS X Edition. The gDNA samples were normalized to the concentration and volume required for the Illumina TruSeq Nano library preparation kits, then sheared to a 450 bp mean insert size using a Covaris LE220 focused-ultrasonicator (Covaris, Brighton, UK). The inserts were ligated with blunt-ended, A-tailed, size-selected TruSeq adapters and enriched using 8 cycles of PCR amplification. The libraries were evaluated for mean peak size and quantity using the Caliper GX Touch, with an HT DNA 1K/12K/HI SENS LabChip and HT DNA HI SENS Reagent Kit (Revvity, Waltham, MA, USA). The libraries were normalized to 5 nM using the GX data, and their actual concentration was established using a Roche LightCycler 480 and a Kapa Illumina Library Quantification kit and Standards (Illumina, Cambridge, UK). The libraries were normalized, denatured, and pooled in groups of eight for clustering and sequencing using a Hamilton MicroLab STAR with the Genologics Clarity LIMS X Edition. Libraries were clustered onto HiSeqX Flow cell v2.5 on cBot2s, and the clustered flow cells were transferred to a HiSeqX for sequencing using a HiSeqX Ten Reagent kit v2.5 (Illumina, Cambridge, UK). Samples were sequenced in paired-end mode with 150 bp read length. The coverage ranged from 19 to 32X.4.4. Mapping and Variant Analysis.

Sequence data processing involved initial quality checking using FASTQC (v.0.11.7), ref. [49] mapping of sequence reads against the Galgal5.0 chicken reference genome (GCA_000002315.3) using the BWA–mem algorithm (v.0.7.15) [50], marking duplicated reads using Picard (v.2.9.2) (https://broadinstitute.github.io/picard/), and Base Quality Score Recalibration of the mapped reads using GATK v3.8 [51]. Significant improvement in accuracy of the base quality scores was observed after applying the GATK recalibration procedure. The GATK-recommended best-practices protocol for ‘Germline Short Variant Discovery’ (https://gatk.broadinstitute.org/hc/en-us/articles/360035535932-Germline-short-variant-discovery-SNPs-Indels-) was used for calling variants. Variant Quality Score Recalibration (VQSR) was applied to minimize false positive calls. A set of ~1 M validated SNPs from a previous study [8] were used as the training set. Significant improvement in the accuracy of the base quality scores was observed after applying the GATK recalibration procedure. The results from recalibration are shown in Supplementary Figure S2. SNPs on autosomes 1 to 33, and the sex chromosomes (W, Z) and the LGE64 linkage group were selected for further analysis. Filtrations were applied based on MAF > 0.05, missing genotype rate (≤10%), and SNP genotype quality (Q > 15).

### 4.4. Genome-Wide Association Study (GWAS)

Plink 1.9 [52] was used to convert vcf files to ped and map format for further analysis. Linkage-disequilibrium pruning was performed in PLINK ver 1.0.7, based on an r^2^ threshold of 0.2 (using options: --indep-pairwise 10 kb 10 0.5), in order to obtain a subset of approximately independently segregating SNPs. After removing the SNPs from chromosome Z (which did not show true population structure like the autosomes), 58,756 independent SNPs were retained. These SNPs were used in multi-dimensional scaling analysis to correct for population stratification and to obtain genome-wide significance thresholds. Relatedness among the samples was estimated based on genome-wide data using the VCFTools (v0.1.16) ‘Relatedness2′ option.

### 4.5. Variant Annotation and Identification of Candidate Genes

Annotation was performed using SnpEff software (v.4.3) [53], based on Ensembl release 86. The annotations for selected variants were checked after liftover (NCBI remap tool) to the updated GRCg6a reference genome (GCA_000002315.5) and VEP (Ensembl Variant Effect Predictor: https://www.ensembl.org/Tools/VEP) were used to verify the effects for both Galgal5 and GRCg6a. Classification of effects by impact on protein function (HIGH, MODERATE, LOW) was performed for all variant annotations [54]. Genes that overlap with the regions of interest were identified with the Ensembl BioMart webtool (https://useast.ensembl.org/info/data/biomart/index.html) based on the Galgal5.0 assembly and the Ensembl Genes 86 annotation database.

### 4.6. Genotyping

Males of recent generations of the Hy-Line elite lines that were relevant to the commercial birds that were sampled were surveyed for SNPs that were identified to be associated with HPAI survival. Detection assays were developed for a total of 236 candidate SNPs that were identified by the GWAS analysis as significantly different between survivors and controls and with prior biological knowledge about genes involved in HPAI resistance. Any assays that did not provide a clear distinction between the two possible alleles, or that had indications of gene duplication, were eliminated, resulting in a final set of 130 validated SNP assays. These 130 SNPs (Supplementary Table S3) were genotyped across four elite pure lines to provide within-line segregation information. These lines were those White Leghorn birds that were related to the survivor samples examined in this study. The single-SNP genotyping was performed utilizing PACE^®^ chemistry (3CR Bioscience Ltd., Harlow, UK), which uses one common and two allele-specific primers. Fluorescence labeling specific to each SNP allele provides for end-point reads to identify the presence of the different alleles. This chemistry is capable of identifying both alleles for the specific SNP, as well as the presence or absence of insertion/deletions [55]. The number of samples tested for segregation ranged from 140 to 180 males per line.

### 4.7. Functional Annotation of Candidate Genes

All genes that overlapped significant regions that were identified in the GWAS were examined for functional enrichment and in pathway analysis, based on the hypothesis that genes that interact in similar biological networks may all be working together to contribute to the resistance phenotype. Pathway analysis was carried out using Qiagen’s Ingenuity Pathway Analysis (IPA) (QIAGEN Inc., https://digitalinsights.qiagen.com/IPA), while functional enrichment was assessed using the WEB-based GEne SeT AnaLysis Toolkit (WebGestalt) [56].

## 5. Conclusions

Samples from commercial chickens that have survived infection with HPAI offer us the unique opportunity to investigate the genetics underlying resilience to HPAI. However, for GWAS, sample numbers were small, and thus the study was admittedly underpowered (odds ratio = 2.001 with 80% power). Resilience to HPAI is also most likely determined by many genes, and so no single gene which could be used to a large effect in direct selection for AI resistance was identified. However, a number of genes with putative association were determined, with some candidates having biological functions that are relevant for antiviral activity. Identification of genomic regions and candidate genes that are potentially associated with HPAI survival in chickens has increased our understanding of HPAI viral pathogenesis, and some of the candidate genes may provide targets for future research, using methods such as gene editing, to determine markers for selective breeding or for improved vaccine development. Future work will validate the signals identified in this study, including analysis of sequence data from additional samples and functional testing of candidate genes in vitro.

## Figures and Tables

**Table 1:** (**A**) SNP effects by putative impact according to SNPEff variant annotations. (**B**) Categories of exonic variant by functional class.

(**A**)		
**Type**	**Genomes = 70**	
	**Count**	**%**
HIGH	5771	0.031
MODERATE	81,717	0.443
LOW	179,100	0.971
MODIFIER	18,176,280	98.555
(**B**)		
**Type**	**Genomes = 70**	
	**Count**	**%**
MISSENSE	81,996	35.02%
NONSENSE	3246	1.39%
SILENT	148,897	63.59%

**Table 2:** Ten SNPs with *p*-values below the genome-wide association threshold in chicken reference genome Galgal5.

Chr	bp	Variant Impact	A1	A2	F_A	F_U	P	Gene	Gene Name
1	176,107,597	Intergenic	T	C	0.2125	0.6481	3.855 × 10^−7^	-	-
1	194,443,048	Upstream gene variant	T	G	0.1364	0.587	5.08 × 10^−7^	APBB1	Amyloid Beta Precursor Protein Binding Family B Member 1
1	194,449,994	Intron variant	G	A	0.183	0.692	5.23 × 10^−8^	SMPD1	sphingomyelin phosphodiesterase
2	149,486,653	Upstream gene variant	G	C	0.065	0.48	6.52 × 10^−8^	NRBP2 (218 bp upstream)	nuclear receptor binding protein 2
5	27,494,876	Intron variant	C	T	0.029	0.426	7.07 × 10^−8^	SYNJ2BP	synaptojanin 2 binding protein
8	6,100,203	Intron variant	C	T	0.08065	0.5192	2.096 × 10^−7^	ADAMTSL2L	ADAMTS like 2-like
10	3,847,722	Intron variant	T	C	0.075	0.52	1.00 × 10^−8^	DAPK2	death associated protein kinase 2
11	17,940,900	Intergenic	T	G	0.02778	0.3929	1.524 × 10^−7^	-	-
11	17,940,906	Intergenic	A	C	0.027	0.393	1.02 × 10^−7^	-	-
20	10,098,517	Intron variant	T	G	0.01562	0.4	1.494 × 10^−7^	ANGPT4	angiopoietin 4

A1/A2—allele 1 and allele 2; F_A—frequency in affected (survivor) birds; F_U—frequency in unaffected (control) samples. P is *p*-value from case–control association test.

**Table 3:** Single-nucleotide polymorphisms based on functional annotation.

Chr	Position bp (GRCg6a)	dbSNP ID	REF	ALT	F_A	F_U	Gene	SNP Functional Impact
1	25,231,313	rs734852275	G	T	0.274	0.646	CAV2	missense_variant
4	32,315,861		C	T	0.363	0.071	NR3C2	missense_variant
9	16,095,724		G	A	0.061	0.38	CHRD	missense_variant
15	3,487,247		T	C	0.241	0.65	RAN	splice_donor_variant and intron_variant

REF—reference allele; ALT—alternative allele; F_A—frequency in affected (survivor) birds; F_U—frequency in unaffected (control) samples. P is *p*-value from case–control association test.

**Table 4:** Significant SNPs identified after imputation.

Chr	bp (Galgal5)	Variant Impact	A1	A2	F_A	F_U	P	Gene	Gene Name
1	173,918,608	intron_variant & non_coding_transcript_variant	A	G	0.4747	0.1974	2.50 × 10^−7^	*ENSGALG00000068037*	LncRNA
1	173,916,044	intron_variant & non_coding_transcript_variant	A	G	0.481	0.2039	2.91 × 10^−7^	*ENSGALG00000068037*	LncRNA
1	168,598,981	intergenic_variant	G	C	0.557	0.2697	2.92 × 10^−7^		
1	173,916,083	intron_variant & non_coding_transcript_variant	G	T	0.4747	0.2039	5.07 × 10^−7^	*ENSGALG00000068037*	LncRNA
1	173,918,500	intron_variant & non_coding_transcript_variant	A	G	0.4747	0.2039	5.07 × 10^−7^	*ENSGALG00000068037*	LncRNA
1	168,599,422	intergenic_variant	A	G	0.5506	0.2697	5.13 × 10^−7^		
1	168,598,342	intergenic_variant	A	G	0.557	0.2763	5.56 × 10^−7^		
1	173,914,795	intron_variant & non_coding_transcript_variant	T	G	0.462	0.1974	7.60 × 10^−7^	*ENSGALG00000068037*	LncRNA
1	173,916,420	intron_variant & non_coding_transcript_variant	T	G	0.462	0.1974	7.60 × 10^−7^	*ENSGALG00000068037*	LncRNA
1	173,918,559	intron_variant & non_coding_transcript_variant	A	C	0.462	0.1974	7.60 × 10^−7^	*ENSGALG00000068037*	LncRNA
1	173,919,150	intron_variant & non_coding_transcript_variant	C	G	0.462	0.1974	7.60 × 10^−7^	*ENSGALG00000068037*	LncRNA
2	22,423,050	intron_variant	G	A	0.0443	0.2566	1.47 × 10^−7^	*AKAP9*	A-Kinase Anchoring Protein 9
2	22,424,983	intron_variant	C	T	0.05063	0.2632	2.33 × 10^−7^	*AKAP9*	A-Kinase Anchoring Protein 9
2	22,425,814	intron_variant	T	C	0.0443	0.25	2.75 × 10^−7^	*AKAP9*	A-Kinase Anchoring Protein 9
2	22,424,305	intron_variant	G	C	0.05063	0.2566	4.32 × 10^−7^	*AKAP9*	A-Kinase Anchoring Protein 9
2	22,424,340	intron_variant	G	A	0.05063	0.2566	4.32 × 10^−7^	*AKAP9*	A-Kinase Anchoring Protein 9
2	22,424,681	intron_variant	G	A	0.05063	0.2566	4.32 × 10^−7^	*AKAP9*	A-Kinase Anchoring Protein 9
2	22,425,953	intron_variant	C	T	0.0443	0.2434	5.12 × 10^−7^	*AKAP9*	A-Kinase Anchoring Protein 9
2	22,425,957	intron_variant	C	T	0.0443	0.2434	5.12 × 10^−7^	*AKAP9*	A-Kinase Anchoring Protein 9
2	22,425,959	intron_variant	C	T	0.0443	0.2434	5.12 × 10^−7^	*AKAP9*	A-Kinase Anchoring Protein 9
5	36,922,525	intron_variant	A	T	0.6203	0.3355	5.28 × 10^−7^	*SLC25A21*	Solute Carrier Family 25- Member 21
15	5,156,114	intron_variant & non_coding_transcript_variant	T	C	0.3038	0.5855	5.94 × 10^−7^	*ENSGALG00000064574*	LncRNA
20	9,369,728	upstream_gene_variant	G	A	0.02532	0.2039	6.76 × 10^−7^	*ENSGALG00000049648*	LncRNA
28	145,697	downstream_gene_variant	T	A	0.3797	0.125	2.67 × 10^−7^	*ENSGALG00000047550*	
28	145,611	downstream_gene_variant	T	C	0.3544	0.1118	4.85 × 10^−7^	*ENSGALG00000047550*	
28	145,315	downstream_gene_variant	G	A	0.3481	0.1118	8.46 × 10^−7^	*ENSGALG00000047550*	

Chr—chromosome; A1/A2—allele 1 and allele 2; F_A—frequency in affected (survivor) birds; F_U—frequency in unaffected (control) samples; P—*p*-value.

**Table 5:** Number of effects by putative impact according to variant annotations for identified INDELs.

Type	n = 70 Samples	
	Count	%
HIGH	80,123	1.087
LOW	14,557	0.198
MODERATE	21,037	0.285
MODIFIER	7,253,373	98.43

**Table 6:** Five INDELs with lowest *p*-values based on the genome-wide association analysis (*n* = 70).

Chr	Position bp (GRCg6a)	Variant	A1	F_A	F_U	A2	P	Gene
		Impact						
10	19,660,768	Intron variant;	T	0.0513	0.537	51 bp	2.35 × 10^−10^	CORO2B; ANP32A
		Upstream gene variant						
19	7,551,566	Intron variant	57 bp	0.6765	0.0385	G	1.56 × 10^−12^	RNFT1
25	2,617,820	Upstream gene variant	C	0.1667	0.8462	CTT	7.11 × 10^−13^	PRCC
25	2,617,813	Upstream gene variant	CCG	0.1765	0.8269	C	1.38 × 10^−12^	PRCC
Z	65,874,960	Intron variant	T	0.65	0.0741	TGTAGGAGAA	3.36 × 10^−11^	PALM2

Chr—chromosome; A1/A2—allele 1 and allele 2; F_A—frequency in affected (survivor) birds; F_U—frequency in unaffected (control) samples; P—*p*-value.

**Table 7:** Number of genotyped-candidate SNPs preselected based on association results and their allele frequencies within the 2017 and 2018 generations of four elite lines.

Chr	N SNPs	Line 1 2017		Line 2 2018		Line 3 2018		Line 4 2018	
		Mean Frequency	N Segregating	Mean Frequency	N Segregating	Mean Frequency	N Segregating	Mean Frequency	N Segregating
1	29	0.44	9	0.50	15	0.52	23	0.30	17
2	11	0.34	5	0.50	9	0.58	4	0.60	9
3	4	0.51	3	0.56	4	0.41	4	0.47	1
4	11	0.35	7	0.46	8	0.49	7	0.53	9
5	3	0.37	2	0.88	3	0.13	2	0.21	2
6	24	0.51	23	0.54	17	0.47	24	0.50	24
7	1	0		0.90	1	1		0.04	1
8	9	0.60	2	0.43	6	0.62	4	0.43	6
9	1	0.29	1	0.18	1	0		0.40	1
10	3	0.69	3	0.62	3	0.85	3	0.52	2
11	1	0		0.90	1	0.19	1	0.05	1
13	1	0.06	1	1		0.32	1	0.82	1
14	5	0.86	4	0.93	4	0.76	4	0.50	5
15	1	0		0.74	1	0.29	1	0.57	1
17	1	0.40	1	1		0.67	1	0.91	1
19	1	0.77	1	0.43	1	0.70	1	0.95	1
20	10	0.52	4	0.33	8	0.60	3	0.66	9
21	1	0		0		0.52	1	0.00	
23	1	0		0.55	1	0		0.01	1
25	2	0.55	2	0.61	2	0.52	2	0.01	1
26	2	0.06	1	0.23	1	0.50		0.46	2
27	1	0.01	1	0.18	1	0.34	1	0.32	1
28	2	0.50	1	0.54	2	0.49	2	0.98	2
33	1	1		1		1		0.03	1
Z	4	0.51	2	0.61	1	0/1		0.48	4

N segregating—number of segregating SNPs in current generation; no threshold for segregation just above 0 and under 1.

## Data Availability

The datasets presented in this article are not publicly available due to the proprietary nature of the data from the commercial birds sequenced. Requests to access the data should be directed to HyLine Int. (Travis Williams—Twilliams@hyline.com).

## Supplementary Materials

The following supporting information can be downloaded at: https://www.mdpi.com/article/10.3390/ijms251810056/s1.
