# Supplementary material for: Candidate Genes Associated with Survival Following Highly Pathogenic Avian Influenza Infection in Chickens
Source: Int J Mol Sci. 2024 Sep 19;25(18):10056. doi: 10.3390/ijms251810056 (PMC11432379; doi:10.3390/ijms251810056)
Supplement: Supplementary file 1 [file ijms-25-10056-s001.zip › ijms-3190098-Supp files.pdf]

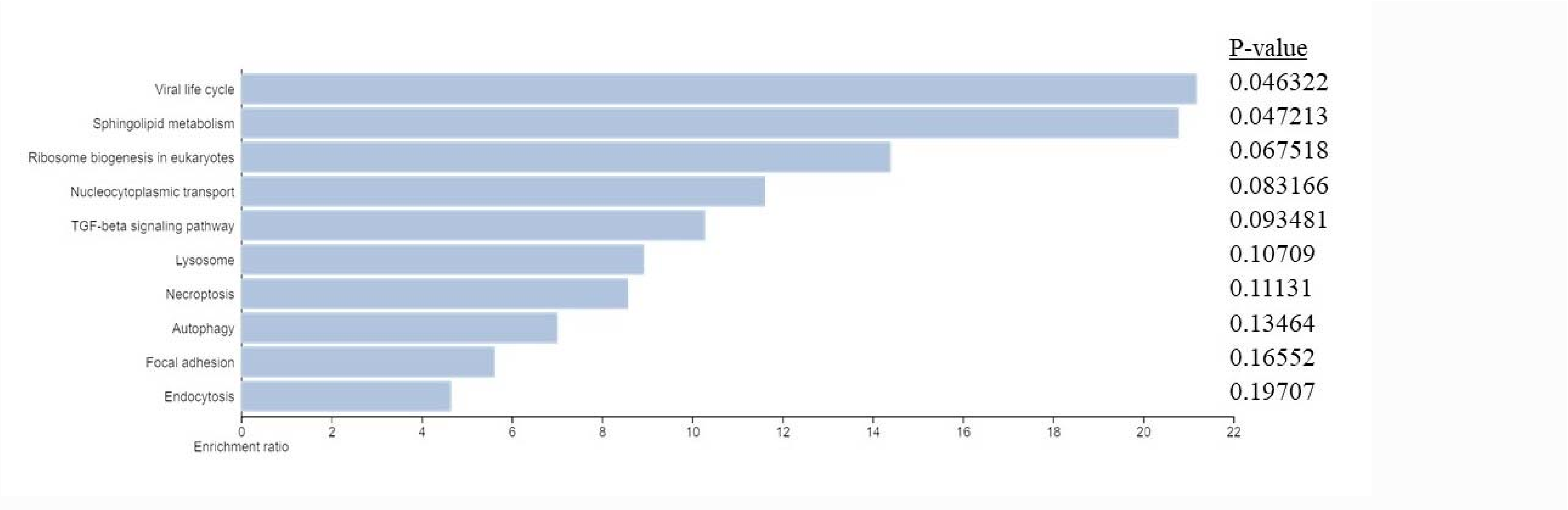

Supplementary Figure S1. Pathway enrichment analysis using WebGestalt. ‘Viral life cycle’ and ‘sphingolipid metabolism’ show p-values <0.05.

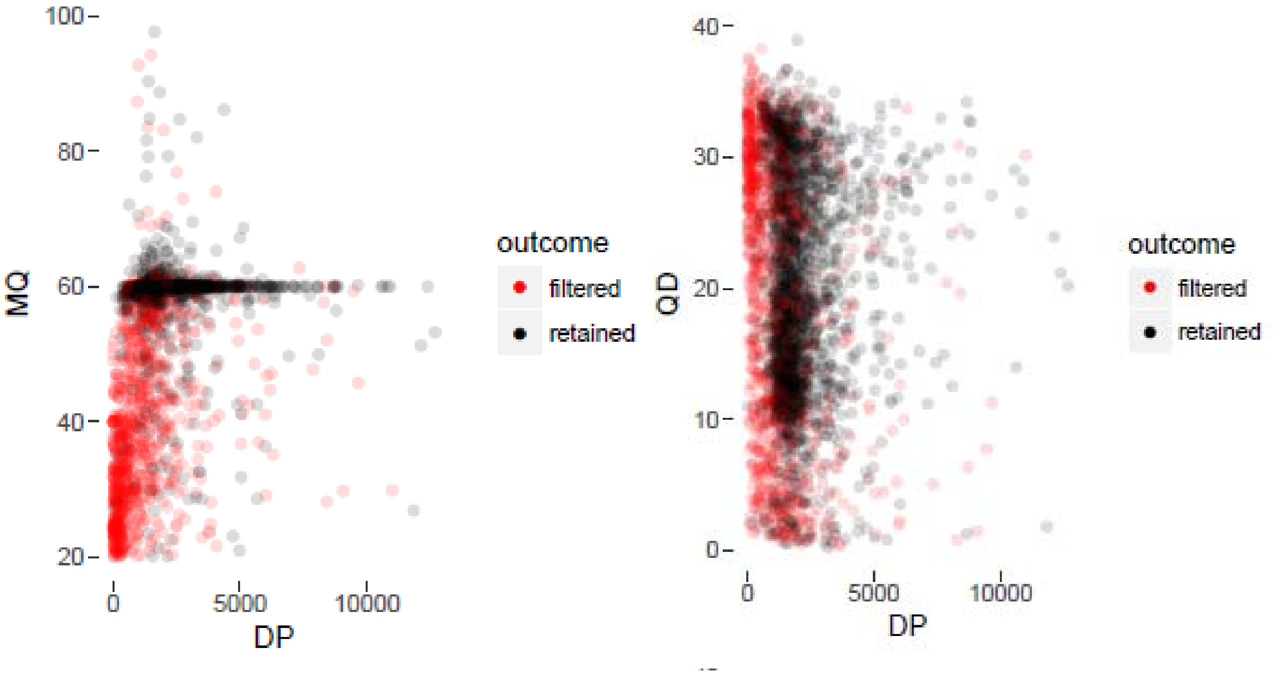

Supplementary Figure S2. Variant recalibration for Mapping Quality (MQ) and Quality by Depth (QD) for all samples according to depth of coverage (DP).

Supplementary Table S1. Correlation of sequence data with 600k genotyping data

Group	N	Mean correlation	Standard deviation
DC	23	0.951	0.025
DS	29	0.920	0.040
MC	5	0.828	0.098
MS	13	0.805	0.062

First letter in group field is an indication of sample origin (company), while the second letter points to cases (S = survivor) or controls (C).

Supplementary Table S2. Genotype concordance and non-reference discrepancy for selected samples after imputation of missing genotypes.

Sample	Mean coverage	% 15X	N	NRD	GC
1-DCHA15	43.8	79.3	104875	2.780	0.984
2-DCHB10	29.1	57.3	104576	4.563	0.975
3-DSHB20	16.9	19.0	103699	8.766	0.951
4-MSHA23	31.8	14.3	101518	15.827	0.916
5-DSHB07	14.2	4.1	99116	18.647	0.903
6-MSHA06	16.4	2.9	87945	25.409	0.885
7-DCHA06	33.3	72.8	104814	4.031	0.977
8-DCHB02	31.6	62.7%	104587	5.468	0.969
9-DSHA14	19.6	25.7%	103642	8.563	0.953
10-DSHA22	25.3	44.9%	104317	5.326	0.970
11-MCHA21	30.3	43.6%	91393	24.629	0.884
12-MSHA32	7.9	3.1%	96936	20.105	0.899

Supplementary Table S3: details of 130 SNPs genotyped across elite lines

SNP	Chr	GRCg6a (bp)	SNP ID	Gene	Strand	Coding	Genic location	Change	Affymetrix SNP ID	Codon	AA substitution
AI2019-090	1	633,435	rs317098996	ENSGALG00000033919	forward	no	intron	T>C			
AI2019-022	1	25,231,313	rs734852275	CAV2	reverse	yes	Exon 1	C>A		GCG>GAG	A25E
AI2019-012	1	45,914,850	rs313133505	intergenic				A>G			
AI2019-079	1	136,293,854	rs315372361	intergenic				A>G			
AI2019-023	1	138,053,495	rs318168487	intergenic				C>G			
AI2019-124	1	140,996,034	rs732238701	intergenic				C>T			
AI2019-084	1	166,501,869	rs732351720	intergenic				T>C			
AI2019-101	1	167,146,830	rs732542523	ELF1	reverse	yes	intron	G>C			
AI2019-105	1	167,287,341	rs315416879	intergenic				T>C			
AI2019-183	1	167,547,923	rs738957933	DGKH	forward	yes	intron	A>G			
AI2019-152	1	167,815,338	rs316337144	intergenic				G>A			
AI2019-153	1	170,145,951	rs316319431	RB1	forward	no	intron	T>A			
AI2019-130	1	170,146,821	rs314245527	RB1	forward	yes	intron	G>A			
AI2019-120	1	170,180,812	rs736884731	intergenic				C>T			
AI2019-102	1	170,387,812	rs735869245	FNDC3A	forward	yes	intron	A>G			
AI2019-142	1	170,445,162	rs740824110	intergenic				G>C			
AI2019-189	1	171,049,836	rs314541350	intergenic				C>A			
AI2019-125	1	171,476,137	rs741041487	ENSGALG00000049809	reverse	no	intron	C>T			
AI2019-040	1	177,435,909	rs316621968	intergenic				C>T			
AI2019-141	1	177,437,025	rs313768711	intergenic				A>T			
AI2019-119	1	177,491,014	rs313144143	ENSGALG00000039958	reverse	yes	intron	T>A			
AI2019-137	1	177,941,198	rs316996507	ENSGALG00000051393	reverse	no	intron	C>T			
AI2019-115	1	177,951,889	rs315418322	intergenic				T>C			
AI2019-194	1	178,088,490	rs731465012	ATP8A2	forward	yes	intron	C>T			
AI2019-180	1	178,089,222	rs14924422	ATP8A2	forward	yes	intron	A>G			
AI2019-046	1	197,402,137	rs733688400	SMPD1	forward	yes	intron	C>G			
AI2019-034	1	197,402,151	rs16732819	SMPD1	forward	yes	intron	T>C			
AI2019-041	1	197,409,098	rs741654070	APBB1	reverse		intron	G>T			

AI2019-188	1	197,502,967	rs315998514	ENSGALG00000017334	reverse	yes	intron	T>C			
AI2019-025	2	476,945	rs313706928	SSPO	forward	no	Exon 28	G>A			
AI2019-104	2	49,237,618	rs731525769	AMPH	reverse	yes	intron	T>C			
AI2019-149	2	102,477,919	rs735776767	MIB1	forward	yes	Exon 1	C>A		TCC>TCA	S224S
AI2019-071	2	116,012,073	rs731216334	intergenic				T>C			
AI2019-159	2	116,947,789	rs14239572	EYA1	reverse	yes	intron	G>A			
AI2019-007A	2	122,579,737	rs735167073	ENSGALG00000041612	reverse	yes	Exon 1	T>G		GAT>GAG	D11E
AI2019-007B	2	122,586,268	rs313109364	LRRCC1	forward	yes	Exon 2	A>G		ATC>GTC	I40V
AI2019-008A	2	124,282,920	rs15152062	NBN	reverse	yes	Exon 11	G>A	AX-75993723	GCA>ACA	A475T
AI2019-009A	2	126,223,442	rs316011897	ENSGALG00000033089	forward	yes	Exon 9	C>T		ACG>ATG	T742M
AI2019-009B	2	126,230,621	rs316598478	ENSGALG00000033089	forward	yes	Exon 15	T>G		CAT>CAG	H1136Q
AI2019-009C	2	126,249,113	rs312446570	ENSGALG00000033089	forward	yes	Exon 33	G>A		GGA>AGA	G2718R
AI2019-121	3	21,880,116	rs313233618	FLVCR1	reverse	yes	intron	A>G			
AI2019-043	3	21,903,461	rs317497766	NSL1	forward	yes	intron	T>C			
AI2019-080	3	21,931,967	rs315057944	intergenic				C>T			
AI2019-110	3	104,562,632	rs736905967	intergenic				C>T			
AI2019-154	4	29,477,839	rs316771098	ENSGALG00000009809	reverse	yes	intron	A>G			
AI2019-026	4	32,315,861	rs312901816	NR3C2	reverse	yes	Exon 2	G>A	AX-76644206	ATG>ATA	M374I
AI2019-004A	4	34,247,658	rs316381850	intergenic				T>C			
AI2019-004B	4	34,247,702	rs739216144	intergenic				A>G			
AI2019-175	4	68,305,279	rs317570998	BEND4	forward	yes	intron	G>A			
AI2019-005A	4	79,253,447	rs741034557	intergenic				G>A			
AI2019-005C	4	79,435,301	rs14494669	intergenic				C>A			
AI2019-177	4	83,866,325	rs314845853	intergenic				T>C			
AI2019-062	4	83,916,573	rs737670141	ENSGALG00000034065	forward	yes	intron	A>G			
AI2019-015	4	84,768,397	rs739052693	intergenic				G>T			
AI2019-114	4	89,132,461	rs16452707	intergenic				G>A	AX-76759207		
AI2019-091	5	330,566	rs315479924	CYB561A3	reverse	yes	Exon 6	G>A		non coding region	
AI2019-160	5	9,121,470	rs16462119	intergenic				T>A			
AI2019-172	5	27,017,984	rs312499835	RGS6	reverse	yes	intron	T>G			

AI2019-010A	6	18,621,052	rs10728407	WASHC2C	reverse	yes	Exon 28	A>G	ATA>ATG	I1127M
AI2019-010B	6	18,636,393	rs13568282	WASHC2C	reverse	yes	Exon 14	A>G	AAA>AGA	K439R
AI2019-010C	6	18,640,070	rs313769058	WASHC2C	reverse	yes	Exon 11	C>T	CCG>CTG	P345L
AI2019-156	6	21,776,505	N/A	intergenic				T>C		
AI2019-126	6	32,961,916	rs80619287	intergenic				C>G		
AI2019-168	6	33,033,324	rs740135195	LHPP	forward	yes	intron	A>G		
AI2019-135	6	33,054,222	rs16566871	LHPP	forward	yes	intron	T>C		
AI2019-185	6	33,082,729	rs732860841	intergenic				G>A		
AI2019-087	6	33,090,261	rs80647945	intergenic				T>A		
AI2019-067	6	33,114,740	rs738909777	ENSGALG00000034013	reverse	yes	intron	C>T		
AI2019-064	6	33,119,106	rs731224305	intergenic				A>G	insertion	
AI2019-056	6	33,138,620	rs315351305	intergenic				T>C		
AI2019-116	6	33,154,348	rs313617118	intergenic				G>C		
AI2019-122	6	33,154,358	rs315138302	intergenic				C>T		
AI2019-065	6	33,196,812	rs736073248	CTBP2	reverse	yes	intron	A>G		
AI2019-176	6	33,196,855	rs740654145	CTBP2	reverse	yes	intron	T>C		
AI2019-171	6	33,198,299	N/A	CTBP2	reverse	yes	intron	T>C		
AI2019-181	6	33,198,841	rs316669079	CTBP2	reverse	yes	intron	G>A		
AI2019-133	6	33,199,268	rs740395423	CTBP2	reverse	yes	intron	C>G		
AI2019-147	6	33,246,139	rs741125686	intergenic				T>A		
AI2019-113	6	33,267,462	rs16080846	intergenic				A>G		
AI2019-146	6	33,324,664	rs13811828	intergenic				A>G		
AI2019-151	6	33,325,221	rs13811825	intergenic				G>A		
AI2019-068	6	33,381,830	rs315254281	intergenic				G>A		
AI2019-190	7	10,035,635	rs318164139	PGAP1	reverse	yes	intron	G>A		
AI2019-157	8	4,634,657	rs314206288	ENSGALG00000002988	reverse	no	intron	A>C		
AI2019-195	8	6,467,475	rs739589558	intergenic						
AI2019-039	8	6,467,484	rs733400733	intergenic				T>C		
AI2019-075	8	6,467,505	rs736773349	intergenic				C>T		
AI2019-058	8	6,467,552	rs736897273	intergenic				G>A		
AI2019-027	8	7,007,066	rs733725693	SEC16B	forward	yes	Exon 18	C>T	GCC>GTC	A739V

AI2019-094	8	16,132,126	rs16632362	intergenic				T>C			
AI2019-163	8	20,805,120	rs15925295	intergenic				G>A			
AI2019-070	8	20,805,184	rs15925297	intergenic				C>G			
AI2019-134	9	16,095,724	rs735267903	CHRD	reverse	yes	Exon1	C>T		TCC>TTC	S29F
AI2019-132	10	3,064,812	rs731050485	intergenic				C>A			
AI2019-060	10	3,287,753	rs313704719	LINGO1	forward	yes	intron	T>C			
AI2019-082	10	3,291,045	rs316756882	intergenic				G>A			
AI2019-096	11	18,840,241	rs317339908	intergenic				G>A			
AI2019-072	13	16,673,462	rs313258192	TCF7	reverse	yes	intron	G>A			
AI2019-011A	14	14,439,555	N/A	ENSGALG00000009205	reverse	yes	Exon 22	G>A		GCA>ACA	A1041T
AI2019-006A	14	14,466,185	rs14086121	ENSGALG00000009205	reverse	yes	intron	C>T			
AI2019-006B	14	14,466,231	rs314022414	ENSGALG00000009205	reverse	yes	intron	G>A			
AI2019-006C	14	14,469,550	rs316965469	ENSGALG00000009205	reverse	yes	intron	C>T	AX-75784230		
AI2019-006D	14	14,482,499	rs738454017	ENSGALG00000009205	reverse	yes	intron	C>T			
AI2019-028	15	3,487,247	N/A	RAN/STX2	reverse/forward	yes/no	intron	n/a			
AI2019-145	17	819,694	rs732453556	FUT7	forward	yes	intron	A>C			
AI2019-029	19	515,054	rs740788614	ENSGALG00000029674	reverse	yes	Exon 1 non coding	G>A			
AI2019-066	20	503,915	rs313708175	GGT7	forward	yes	intron	A>G			
AI2019-117	20	3,754,552	rs15171495	intergenic				A>G			
AI2019-128	20	5,166,230	rs15172762	MATN4	forward	yes	intron	A>G			
AI2019-166	20	9,242,982	rs317096948	NPBWR2	forward	yes	Exon 3	C>T		non coding region	
AI2019-167	20	9,243,540	rs314192817	NPBWR2	forward	yes	intron	G>C			
AI2019-098	20	9,245,437	rs740857434	NPBWR2	forward	yes	intron	T>C			
AI2019-112	20	9,249,927	rs731149049	NPBWR2	forward	yes	intron	C>T			
AI2019-129	20	9,250,560	N/A	NPBWR2	forward	yes	intron	G>C and G>A			
AI2019-059	20	9,895,053	rs735597735	intergenic				A>G			

AI2019-038	20	9,897,430	rs739976344	ANGPT4	forward	yes	intron	G>C and G>T			
AI2019-139	21	2,845,888	N/A	intergenic				T>C			
AI2019-031	23	5,108,887	rs736777049	TINAGL1	forward	yes	Exon 3	C>G		GAC>GAG	D136E
AI2019-179	25	3,010,644	rs741656758	PYGO2	reverse	yes	Exon 2	T>C		non coding region	
AI2019-127	25	3,024,320	rs734268699	FLAD1	forward	yes	Exon 5	C>T		TCT>TTT	S572F
AI2019-182	26	1,692,328	rs733345358	intergenic				A>G			
AI2019-083	26	2,722,608	N/A	intergenic				T>C and T>G			
AI2019-048	27	6,504,770	rs13802938	SP2	reverse	yes	intron	T>C	AX-76364645		
AI2019-081	28	3,552,690	N/A	ENSGALG00000041897	reverse	yes		G>A			
AI2019-170	28	3,995,476	rs739336808	FKBP8	forward	yes	intron	A>G			
AI2019-097	33	7,149,027	N/A	KMT2D	forward	yes	intron	G>C			
AI2019-150	Z	22,406,442	rs15713094	ENSGALG00000050556	forward	no	intron	A>C	AX-77202476		
AI2019-103	Z	22,482,221	N/A	DMGDH	forward	yes	intron	C>T			
AI2019-164	Z	42,414,710	N/A	intergenic				C>T			
AI2019-047	Z	51,276,580	N/A	intergenic				T>C			
